# Possible Outbreak of Streptomycin-Resistant *Mycobacterium tuberculosis* Beijing in Benin

**DOI:** 10.3201/eid1507.080697

**Published:** 2009-07

**Authors:** Dissou Affolabi, Frank Faïhun, N’Dira Sanoussi, Gladys Anyo, Isdore Chola Shamputa, Leen Rigouts, Luc Kestens, Séverin Anagonou, Françoise Portaels

**Affiliations:** Laboratoire de Référence des Mycobactéries, Cotonou, Benin (D. Affolabi, F. Faïhun, N. Sanoussi, S. Anagonou); Institute of Tropical Medicine, Antwerp, Belgium (D. Affolabi, G. Anyo, I.C. Shamputa L. Rigouts, L. Kestens, F. Portaels); 1 Current affiliation: National Institutes of Health, Bethesda, Maryland, USA.

**Keywords:** Outbreak, Beijing strain, Mycobacterium tuberculosis, tuberculosis and other mycobacteria, bacteria, Benin, dispatch

## Abstract

Using geographic information system and molecular tools, we characterized a possible outbreak of tuberculosis caused by *Mycobacterium tuberculosis* Beijing strain in 17 patients in Cotonou, Benin, during July 2005–October 2006. Most patients lived or worked in the same area and frequented the same local drinking bar. The isolates were streptomycin resistant.

In a previous survey aimed at investigating the genetic biodiversity of *Mycobacterium tuberculosis* in Cotonou, Benin (*1*), we observed a higher prevalence of strains belonging to the Beijing genotype than has been reported in other studies in West and Central Africa (*2*–*4*). In that survey, we applied the results of spoligotyping and typing using a 12-loci mycobacterial interspersed repetitive unit–variable number tandem repeat (MIRU-VNTR) profile to identify the genetic lineage of the strains. In the study described here, we further investigated the identified Beijing strains by characterizing them with the more discriminatory set of 24-loci MIRU-VNTR (*5*). We also mapped the residences and workplaces of the patients by using geographic information system (GIS) technology.

## The Study

From July 2005 through October 2006, a survey was conducted on 194 isolates of *M. tuberculosis* obtained from 194 patients with pulmonary tuberculosis (TB) (1 isolate per patient) (*1*). Patients were recruited from the National Hospital for Pneumology and Phtisiology in Cotonou, Benin, where most TB patients from the area are treated. Cotonou is the largest city in Benin, with a population of 655,000 in 2002 and an area of 79 km^2^.

All patients gave informed consent. The study was approved by the National Tuberculosis Program Board of Benin.

Among these 194 isolates, 17 belonged to the Beijing ST1 family and exhibited the same 12-loci MIRU-VNTR pattern (223325163533). One isolate showed additional alleles at many loci. The median age of the patients infected with the *M. tuberculosis* strains belonging to the Beijing genotype (28 years) was similar to that of patients from the general survey (30 years).

Demographic data that included date of birth, age, sex, and places of residence and work were collected from each patient. Geo-coordinates of each patient’s residence and workplace were obtained by using the Global Positioning System (GPS) and mapped with the ArcView 3.2 software (ESRI, Redlands, CA, USA). We also sought to map a place habitually frequented by the patients.

Blood samples for HIV testing were collected from each patient. HIV testing was performed by using an ELISA. Seropositive samples were confirmed by a discriminatory HIV1/2 test (Genie II HIV1/HIV2; Bio-Rad, Marnes-la-Coquette, France).

One sputum sample from each patient was decontaminated by using the modified Petroff method and cultured in manual Mycobacteria Growth Indicator Tube (MGIT) (*6*) and on Löwenstein-Jensen (LJ) medium. All isolates were identified as *M. tuberculosis* complex by the para-nitrobenzoic acid method and tested for drug susceptibility against rifampin, isoniazid, streptomycin, and ethambutol by using the proportion method on LJ medium at the following respective concentrations: 40 µg/mL, 0.2 µg/mL, 4 µg/mL, and 2 µg/mL (*7*,*8*).

DNA was extracted by boiling a suspension of 2 drops of MGIT-positive cultures in 300 µL of 10 mmol/L Tris-HCl and 1 mmol/L EDTA, pH 8.0 (1× Tris-EDTA) for 5 minutes. MIRU-VNTR typing was performed at Genoscreen (Lille, France) by amplifying each of the 24 independent loci, and results were combined into digit allelic profiles (*5*).

All patients discussed here were born in Benin and had lived in the country since birth. How they became infected with the *M. tuberculosis* Beijing strain is unclear. However, because some inhabitants of Cotonou are immigrants from the Asian continent, this strain could have been brought into the country by migrant residents of the community.

In total, 6 (35%) of 17 patients were HIV-1 seropositive, and the remaining 11 patients (65%) were HIV seronegative. In contrast with the results in the initial survey, the M. tuberculosis Beijing strain was more likely to be isolated from HIV seropositive patients than from those who were HIV seronegative (21 [12%] of 173 (p = 0.024).

Of the 17 *M. tuberculosis* isolates belonging to the Beijing genotype, drug susceptibility testing results were available for 16. All isolates from the 16 patients were resistant to streptomycin but susceptible to isoniazid, rifampin, and ethambutol. Of the remaining 177 isolates from the same survey, drug susceptibility testing was available for 127, of which only 13 (10.2%) were resistant to streptomycin. The *M. tuberculosis* Beijing strains in this survey were more likely to be resistant to streptomycin than were their non-Beijing counterparts (p<0.001*)*. Other researchers have suggested that *M. tuberculosis* Beijing strains may be associated with drug resistance (*9*) and that the rapid spread of Beijing strains in some settings suggests an intrinsic virulence of this family (*10*).

The combination of double alleles in several MIRU-VNTR loci of 1 isolate suggests a mixed infection (*11*). The other 16 strains showed identical profiles in the 24 MIRU-VNTR set: loci 154, 424, 577, 580, 802, 960, 1644, 1955, 2059, 2163b, 2165, 2347, 2401, 2461, 2531, 2687, 2996, 3007, 3171, 3192, 3690, 4052, 4156, and 4348 with the following profile: 244233342xx4425163353723. No amplification was achieved for loci 2163b and 2165, despite 2 rounds of PCR for all 16 isolates. This common failure to amplify loci 2163b and 2165, which are close in the *M. tuberculosis* chromosome, might be explained by a chromosomal deletion in the strain responsible for this possible outbreak. Having a chromosomal deletion supports the hypothesis that all patients were infected by the same strain. However, other possible explanations such as nucleotide polymorphisms in the sequence complementary to PCR primers could not be excluded. Many studies have reported pseudo-outbreaks caused by laboratory cross-contamination (*12*,*13*). This is not likely in the present study because almost all the specimens for primary culture were processed on different days over several months.

Mapping of patients’ residences and workplaces showed that many lived or worked (or both) in the Xwlacodji area of Cotonou (Figure). To further investigate this spatial cluster and a possible link between patients, we mapped a place they habitually frequented and found that most patronized the same drinking bar (Figure). Ten of the patients either lived or worked near the bar (<300 m) or regularly visited it. Although no epidemiologic link was evident between these patients and the remaining 7 patients, most of the latter were motorcycle taxi drivers and regularly moved from place to place.

**Figure:**
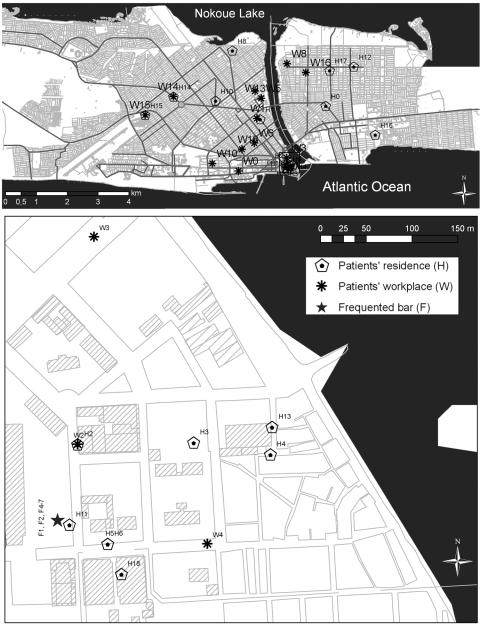
Maps showing residences and workplaces of *Mycobacterium tuberculosis* patients in Xwlacodji, Cotonou, Benin, 2005–2006.

Without GPS we could not have identified the geographic cluster of these patients with Beijing strains in Xwlacodji. The residents are poor, and overcrowding creates conditions favorable for transmission of diseases from person to person and rapid spread of the resistant *M. tuberculosis* Beijing strain.

## Conclusions

We cannot completely exclude the unlikely possibility that the patients were infected by different strains with the same MIRU-VNTR pattern. However, the 17 strains’ identical 24-loci MIRU-VNTR profile, with a probable deletion of the same 2 loci; the identical drug susceptibility pattern (monoresistant to streptomycin); and the fact that most patients resided in the same community as determined by GIS strongly suggest that these strains are part of an outbreak.

Generally, molecular tools are used to study TB transmission and to suggest possible outbreaks. Although molecular tools can help identify an outbreak, they cannot localize it. GIS has rarely been used in health systems, particularly in resource-poor countries such as Benin (*14*). GIS tools might be dispensable in industrialized countries where streets and houses are properly mapped and pinpointing specific addresses is generally sufficient to localize an outbreak. In many low-income settings, however, streets and houses are not properly numbered and mapped, and using GPS is necessary to gain access to accurate geo-coordinates and localize such outbreaks. In the absence of genetic methods, differentiating cases from the same route of transmission from others is difficult. However, GIS can help TB control practitioners identify areas with aggregate cases so they can institute appropriate measures to control the disease.
